# A Rare Cause of Cough: Tracheobronchial Myxoid Spindle Cell Lipoma

**DOI:** 10.1155/2020/9727281

**Published:** 2020-05-30

**Authors:** Jose A. Rodriguez, Christopher R. Weil, Jose F. Ramirez

**Affiliations:** ^1^Department of Internal Medicine, Memorial Healthcare System, Pembroke Pines, FL, USA; ^2^Department of Radiation Oncology, University of Utah, Salt Lake City, UT, USA; ^3^Integrated Sleep Care, Miramar, FL, USA

## Abstract

**Background:**

Endobronchial lipomas are a particularly rare form of benign lung neoplasms, with an incidence of 0.1-0.4%. They are often clinically silent, though present with nonspecific symptoms that can result in extensive workup or significant delay prior to diagnosis, as presented in this case. *Case Presentation*. A 61-year-old male presented with chronic productive cough and occasional dyspnea and a 10-year history of clinically diagnosis of intermittent, exercise-induced asthma, nonresponsive to bronchodilators, and with normal pulmonary function tests. A chest ray showed a band-like opacity in the right middle lobe of the lung and a CT of the chest showed right lung atelectasis with a soft tissue mass in the right main bronchus. The patient underwent bronchoscopy with biopsy, which demonstrated an endobronchial myxoid spindle cell lipoma. The lesion was subsequently removed by a bronchoscopic snare. The patient's asthmatic-like symptomatology resolved after the mass was excised.

**Conclusion:**

Though rare, endobronchial lipomas characteristically present with nonspecific signs and symptoms and thus can be easily mistaken for other medical conditions, delaying diagnosis and prolonging symptoms.

## 1. Introduction

Benign tumors of the lung are uncommon, representing less than 2% of lung neoplasms [1]. Endobronchial lipomas are an especially rare subset of benign lung neoplasms, with an incidence estimated at 0.1-0.4% of all lung tumors and 1.4-13% of all benign lung tumors [2]. Endobronchial lipomas have been associated with obesity and smoking and have been previously reported to present with cough, sputum production, hemoptysis, and dyspnea [3, 4]. They are often clinically mistaken for asthma that does not respond to bronchodilator therapy, resulting in delay of appropriate diagnosis and prolongation of symptoms.

We present a male patient who had been receiving treatment for asthma for the past several years with persistent cough and dyspnea who on CT of the chest and bronchoscopy, and further histopathology was found to have an endobronchial myxoid spindle cell lipoma.

## 2. Case Presentation

**Figure 1:**
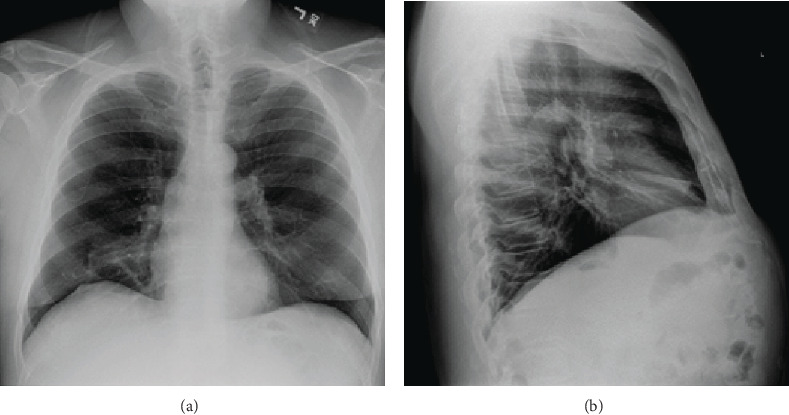
Poster anterior and lateral chest X-ray: right middle lobe infiltrate, band-like in shape, overlying the anterior cardiac contour.

A 61-year-old male, nonsmoker, with a past medical history of ulcerative colitis, dyslipidemia, and exercise-induced asthma, presented to the clinic for evaluation of multiple-year history of chronic cough productive of white sputum with associated occasional dyspnea on exertion, nonresponsive to inhalers. Family history pertinent for a father with lung cancer at age 70. The patient had no identifiable acute or chronic toxigenic exposure. Vital signs, including SpO2, were within normal limits, and physical examination was unremarkable. Spirometry performed six months prior demonstrated normal FVC, FEV1, FEV1/FVC ratio, and DLCO. Posteroanterior and lateral chest X-ray demonstrated a thin band-like opacity overlying the cardiac contour suggesting atelectasis of the right middle lobe (Figure 1).

**Figure 2:**
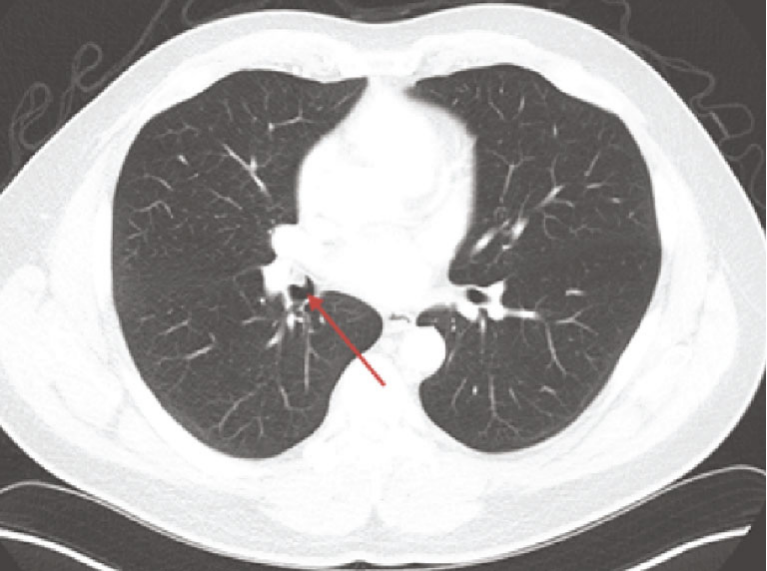
CT chest, axial view: red arrow denoting a 15 mm intraluminal, lobulated exophytic soft lesion at the origin of the right middle lobe.

**Figure 3:**
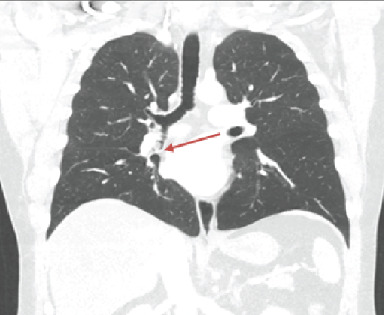
CT chest, coronal view: red arrow denoting another view of the exophytic soft tissue lesion.

**Figure 4:**
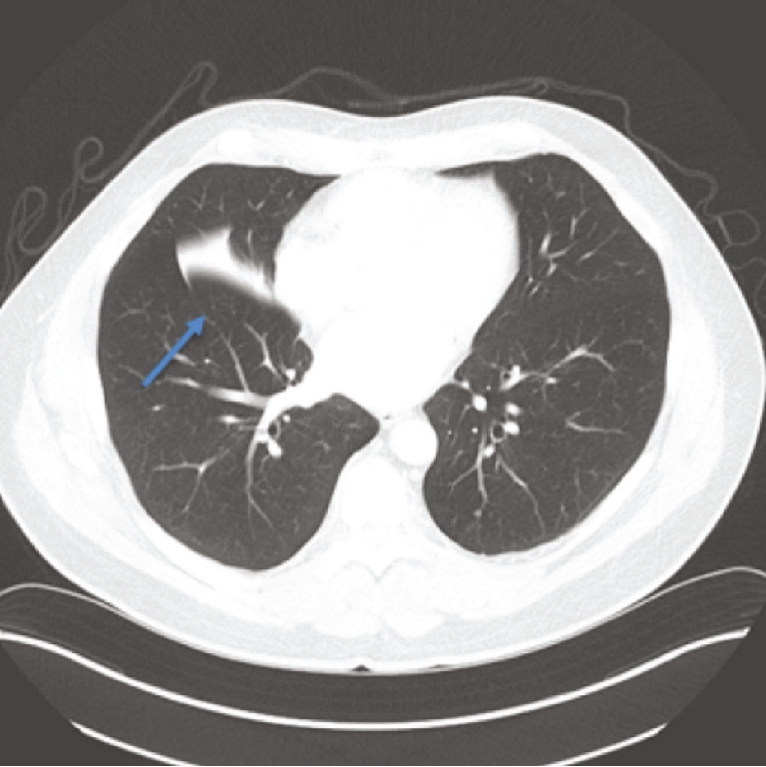
CT chest, axial view: blue arrow denoting an opacity throughout much of the right middle lobe.

Noncontrast CT of the chest showed an endobronchial soft tissue mass at the origin of the right middle lobe bronchus with postobstructive right middle lobe collapse (Figures 2-4).

**Figure 5:**
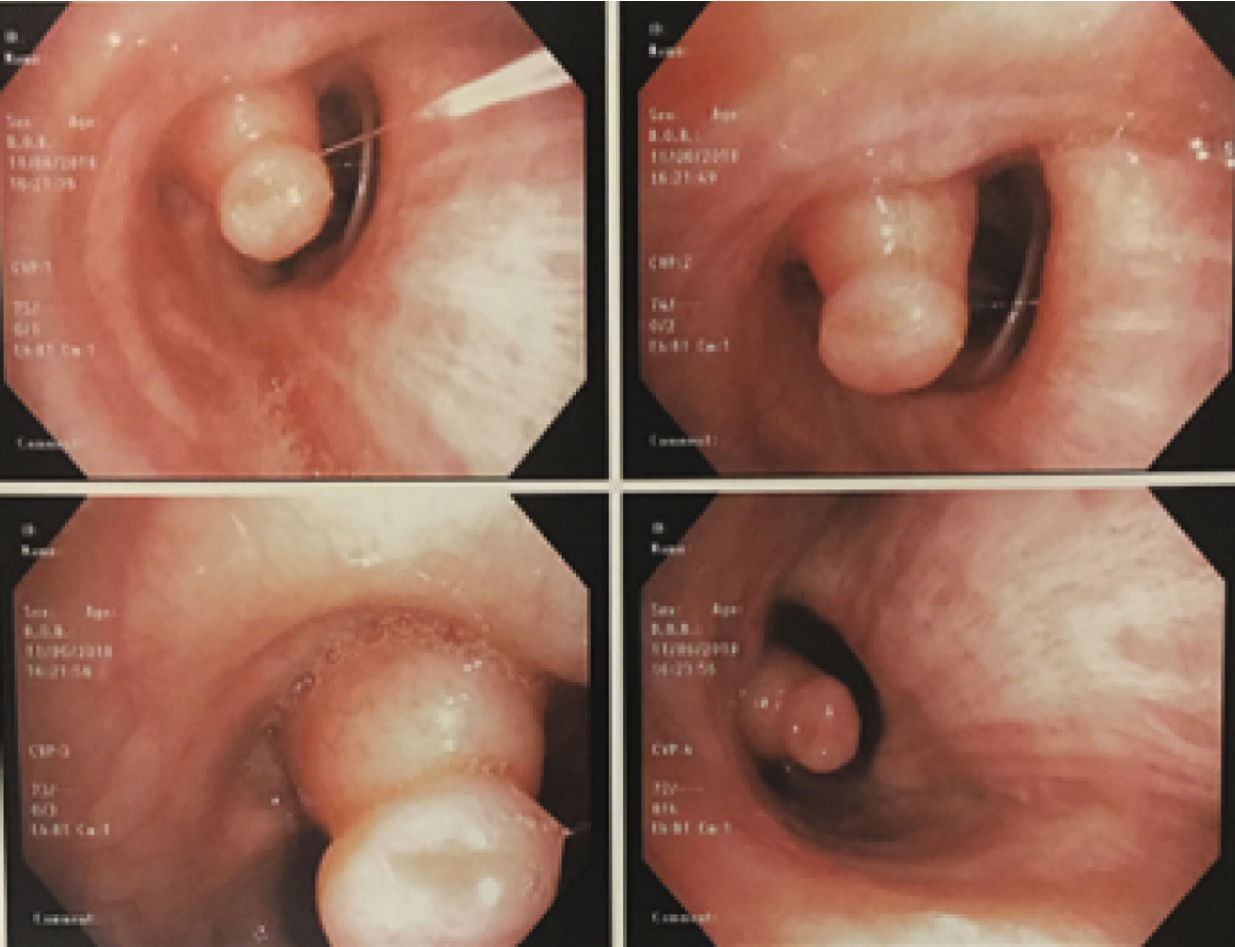
Flexible bronchoscopy: occlusion of the right middle lobe by a 1 × 1 cm sessile lesion.

A flexible bronchoscopy was performed, showing an exophytic, sessile polypoid lesion attached to the medial wall of the right middle lobe origin causing a significant luminal occlusion (Figure 5).

**Figure 6:**
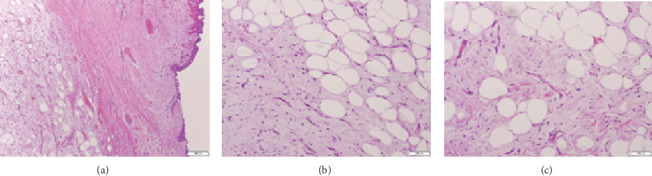
Excisional biopsy of the right middle lobe lesion showing myxoid respiratory tissue with mature adipocytes surrounded by a thick capsule without invasion (a). Characteristic spindle cells with ropey/wavy collagen with interspersed mature adipocytes (b, c), suggestive of a myxoid spindle cell lipoma.

Endobronchial biopsies were taken, with pathology reporting lipoid fragments of the respiratory mucosa and submucosal adipose tissue with mild inflammation and reactive changes. The patient underwent a second flexible bronchoscopy with snaring forceps for a second biopsy; complete removal of the lesion was performed with pathology reporting endobronchial myxoid spindle cell lipoma (Figure 6).

Postbronchoscopy chest X-ray demonstrated complete resolution of the right middle lobe collapse. On follow-up, the patient reported complete resolution of his chronic productive cough as well as the dyspnea negating need for further pulmonary function tests.

## 3. Discussion

Endobronchial lipomas are very unusual tumors originating from adipose tissue present normally in the tracheobronchial wall, appearing pale yellow to whitish-gray in color with smooth round surface. Most of them are found in middle-aged adults with a 90% male predominance [3, 4]. Symptoms are nonspecific and result from a fixed bronchial obstruction, typically presenting with a cough or asthma-like symptoms that are not responsive to bronchodilators, and with normal PFTs [5]. Our patient's main symptom was cough with some occasional dyspnea, since there was no complete central airway occlusion.

Chest X-ray films are not diagnostic but, as in this case, may show atelectasis with an anatomically confined obstructive pneumonia if there is sufficient airway obstruction. A noncontrast CT scan of the chest may reveal low-density soft tissue mass in large bronchi [6].

Given the wide variety of malignant and benign neoplasms, which often have similar gross appearance on bronchoscopy, a broad differential should be entertained prior to biopsy. The most reliable methods for confirming diagnosis are bronchoscopy with bronchial biopsy or excision. Local resection for symptomatic relief is usually accomplished with simple transbronchoscopic excision or bronchotomy, though in rare cases with significant irreversible lung injury, lobectomy or pneumonectomy have been required [7]. As in our case, all symptomatology resolved after the excision of the lesion.

Spindle cell lipoma is an uncommon variant of lipoma, characterized by a circumscribed mass of mature adipocytes and small uniform spindle cells. These are typically found in male patients between the age of 40 to 60, presenting as a subcutaneous mass of the posterior trunk, neck or shoulder, or as a cutaneous lesion in the thigh, pelvis, face, or oral cavity [8]. Tracheobronchial myxoid spindle cell lipoma represents a very atypical location for this type of lesion.

## 4. Conclusion

As typified in this case, endobronchial soft tissue neoplasms are rare but should be considered on the differential in patients with a long history of asthma-like symptoms that are not responsive to medical therapy. Accurate diagnosis is important, as simple resection can lead to complete resolution of pulmonary symptoms.
